# The novel POSEIDON stratification of ‘Low prognosis patients in Assisted Reproductive Technology’ and its proposed marker of successful outcome

**DOI:** 10.12688/f1000research.10382.1

**Published:** 2016-12-23

**Authors:** Peter Humaidan, Carlo Alviggi, Robert Fischer, Sandro C. Esteves

**Affiliations:** 1 Fertility Clinic, Skive Regional Hospital, Skive, Denmark; 2 Faculty of Health, Aarhus University, Skive, Denmark; 3 Department of Neuroscience, Reproductive Science and Odontostomatology, University of Naples Federico II, Naples, Italy; 4 Fertility Center Hamburg, Hamburg, Germany; 5 ANDROFERT, Andrology & Human Reproduction Clinic, Campinas, Brazil

**Keywords:** Assisted Reproductive Technology, Diagnosis, Gonadotropins, Group POSEIDON, Ovarian stimulation, Embryo aneuploidy, Poor ovarian response, Prognosis.

## Abstract

In reproductive medicine little progress has been achieved regarding the clinical management of patients with a reduced ovarian reserve or poor ovarian response (POR) to stimulation with exogenous gonadotropins -a frustrating experience for clinicians as well as patients. Despite the efforts to optimize the definition of this subgroup of patients, the existing POR criteria unfortunately comprise a heterogeneous population and, importantly, do not offer any recommendations for clinical handling. Recently, the POSEIDON group ( **P**atient- **O**riented **S**trategies **E**ncompassing **I**ndividualize **D O**ocyte **N**umber) proposed a new stratification of assisted reproductive technology (ART) in patients with a reduced ovarian reserve or unexpected inappropriate ovarian response to exogenous gonadotropins. In brief, four subgroups have been suggested based on quantitative and qualitative parameters, namely, i. Age and the expected aneuploidy rate; ii. Ovarian biomarkers (i.e. antral follicle count [AFC] and anti-Müllerian hormone [AMH]), and iii. Ovarian response - provided a previous stimulation cycle was performed. The new classification introduces a more nuanced picture of the “low prognosis patient” in ART, using clinically relevant criteria to guide the physician to most optimally manage this group of patients. The POSEIDON group also introduced a new measure for successful ART treatment, namely, the ability to retrieve the number of oocytes needed for the specific patient to obtain at least one euploid embryo for transfer. This feature represents a pragmatic endpoint to clinicians and enables the development of prediction models aiming to reduce the time-to-pregnancy (TTP). Consequently, the POSEIDON stratification should not be applied for retrospective analyses having live birth rate (LBR) as endpoint. Such an approach would fail as the attribution of patients to each Poseidon group is related to specific requirements and could only be made prospectively. On the other hand, any prospective approach (i.e. RCT) should be performed separately in each specific group.

The management of patients with an impaired ovarian reserve or poor ovarian response (POR) to exogenous gonadotropin stimulation has challenged reproductive specialists for decades. Apart from limited understanding of the pathophysiology, wide heterogeneity exists in the definition of the poor responder patient as well as overall disappointing outcomes in assisted reproductive technology (ART) ( Papathanasiou *et al.*, 2016).

The POSEIDON group ( **P**atient- **O**riented **S**trategies **E**ncompassing **I**ndividualize **D O**ocyte **N**umber) was recently established to focus specifically on the diagnosis and management of low prognosis patients ( Poseidon Group, 2016). Composed by reproductive endocrinologists and reproductive medicine specialists from 7 countries [Carlo Alviggi (Italy), Claus Y. Andersen (Denmark), Klaus Buhler (Germany), Alessandro Conforti (Italy), Giuseppe de Placido (Italy), Sandro C. Esteves (Brazil), Robert Fischer (Germany), Daniela Galliano (Spain), Nikolaos P. Polyzos (Belgium), Sesh K. Sunkara (United Kingdom), Fillipo M. Ubaldi (Italy), and Peter Humaidan (Denmark)] with long-standing clinical and/or research experience, the POSEIDON group in an opening paper proposed a new stratification to classify infertility patients with a reduced ovarian reserve or unexpected inappropriate ovarian response to exogenous gonadotropins ( Poseidon Group, 2016). In brief, four subgroups have been suggested based on quantitative and qualitative parameters, namely, i. Age and the expected aneuploidy rate; ii. Ovarian biomarkers (i.e. antral follicle count [AFC] and anti-Müllerian hormone [AMH]), and iii. Ovarian response - provided a previous stimulation cycle was performed ( Figure 1). The POSEIDON group also introduced a new measure for successful ART treatment, namely, the ability to retrieve the number of oocytes needed for the specific patient to obtain at least one euploid embryo for transfer.

**Figure 1.**
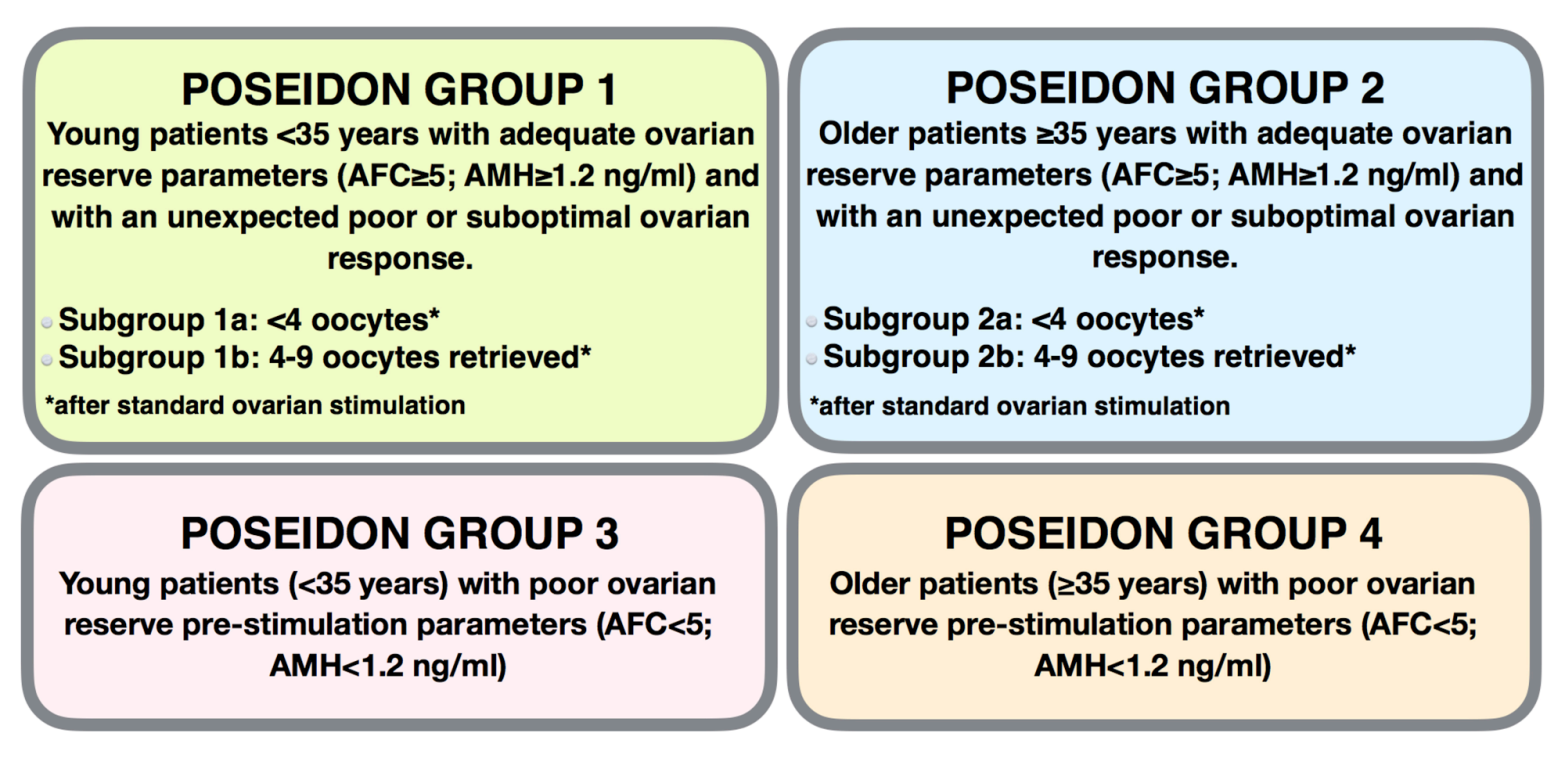
Four groups of ‘low prognosis patients’ in assisted reproductive technology according to the POSEIDON’s stratification based on oocyte quantity and quality. AFC: antral follicle count; AMH: anti-Müllerian hormone. Adapted with permission from Elsevier; Poseidon Group (Patient-Oriented Strategies Encompassing IndividualizeD Oocyte Number)., Alviggi C, Andersen CY, Buehler K, Conforti A, De Placido G, Esteves SC, Fischer R, Galliano D, Polyzos NP, Sunkara SK, Ubaldi FM, Humaidan P. A new more detailed stratification of low responders to ovarian stimulation: from a poor ovarian response to a low prognosis concept. Fertil Steril. 2016 Jun;105(6):1452–3.

Following its publication earlier this year ( Poseidon Group, 2016), the POSEIDON stratification system has sparked interest among infertility practitioners. Here, we expand the discussion as to why the new concept has been proposed, providing new and important information as below.

First, it is clear that the major players involved in the complex POR equation are not fully satisfied with the existing classification criteria. Taking the scholarly perspective, for instance, until now more than 70 randomized controlled trials (RCTs) compared interventions in poor responders using a wide range of definitions, including the most recent Bologna criteria ( Ferraretti *et al.*, 2011; Papathanasiou *et al.*, 2016). Among the trials registered in www.clinicaltrials.gov until November 2016, 44 were specific to POR. However, analyzing the results of completed trials and the published literature, the overall conclusion is that there is insufficient evidence to support the routine use of any particular intervention for POR. Thus, data indicate that the current classification criteria have been unable to discriminate patient subsets within the POR population who could benefit from specific interventions ( Nagels *et al.*, 2015; Pandian *et al.*, 2010; Papathanasiou *et al.*, 2016). A possible explanation is that the analysis of whole populations of POR with different baseline characteristics and, therefore, different prognosis in a given RCT may dilute the effect size.

Along the same lines, but taking the perspective of the clinician, a recent international survey showed that the most frequently used criterion to identify POR was the “number of follicles produced”, which surprisingly has been rarely included in the scholarly definition of POR ( Patrizio *et al.*, 2015). Moreover, due to the absence of efficient remedies, most practices do not use an evidence-based treatment for this category of patients ( Patrizio *et al.*, 2015). Lastly, according to the standpoint of the patient, RESOLVE ( www.RESOLVE.org), a non-for profit patient organization dedicated to providing education to couples suffering from infertility, classifies POR as women who require large doses of medication and who produce less than an optimal number of oocytes. This indicates that patients themselves have introduced a new element into the already complex POR equation, namely, suboptimal response to ovarian stimulation.

Secondly, it is important to further discuss the issue of quantity versus quality regarding oocytes. It is difficult to deny that counting the number of oocytes retrieved or estimating their numbers using ovarian biomarkers may not be sufficient for clinical management. Equally important is the age-related decrease in oocyte quality, which largely depends on chromosomal abnormalities occurring prior to meiosis II ( Sakakibara *et al.*, 2015). Despite recognizing that other biochemical processes are also relevant to oocyte quality, the genetic competence of the oocyte is paramount as it affects the implantation potential of the resulting embryo. For instance, blastocyst euploidy rates of about 60% are observed in younger women (<35 years of age) undergoing ART whereas these numbers fall to 30% or lower in patients aged 40–42 ( Ata *et al.*, 2012). As a result, the age-related embryo aneuploidy rate dramatically changes the prognosis of women with the same oocyte yield as well as those with different oocyte yields.

Lastly, and most importantly we wish to stress the new POSEIDON marker of successful outcome, i.e., the ability to retrieve the number of oocytes necessary to achieve at least one euploid embryo for transfer in each patient. We strongly believe this represents a more pragmatic endpoint for clinicians providing care to infertility patients. Furthermore, it opens the possibility of developing prediction models to help clinicians counsel and set patient expectations and establish a working plan to reduce the time-to-pregnancy (TTP). This is essential to avoid any misunderstanding regarding the POSEIDON concept, as the intention of the concept is to help guide clinicians through the medical management, and as such it should not to be used in retrospective analyses having live birth rate (LBR) as an endpoint.

While LBR is more appropriate for counseling purposes and designing RCTs, the POSEIDON concept is based on (i) a better stratification of women with "low prognosis" in ART, and (ii) individualized therapeutic approaches in each group, having as endpoint the number of oocytes required to have at least one euploid embryo for transfer in each patient. Essentially, the POSEIDON concept was designed to offer a practical endpoint to clinicians as it may help set a clear goal for management.

Obviously, retrospective analyses of previously structured databases can match patients to fit into POSEIDON subgroups. As an example, from an existing database (pre-POSEIDON) one might analyze the LBR of women >=35 years with low ovarian reserve (i.e., POSEIDON group 4). However, assuming commonly reported metaphase II rates (e.g. 75%), 2PN fertilization rates (e.g. 70%), blastulation rates (e.g. 45%), and blastocyst euploidy rates (e.g. 50%), approximately 12 oocytes are needed to obtain at least one euploid blastocyst for transfer in a given 36 year old patient. Nevertheless, it is unlikely that this hypothetical patient was treated according to the POSEIDON concept, using an individualized therapeutic plan, based on the number of oocytes to obtain at least one euploid blastocyst. Hence, any analysis, using LBR as an endpoint to be valid should ensure that patients were prospectively stratified as per POSEIDON groups and treated with the mindset of achieving the proposed POSEIDON marker of success.

In conclusion, in comparison with previously suggested models to define POR patients from a rigid standpoint and without any clinical guidance, the POSEIDON concept contemplates clinical recommendations with a new pragmatic endpoint, the number of oocytes needed to obtain one euploid embryo for transfer in each patient. We see this novel initiative as an important working – and counseling tool for the ART specialist who handles the low prognosis patient.
