# Supplementary material for: The novel POSEIDON stratification of ‘Low prognosis patients in Assisted Reproductive Technology’ and its proposed marker of successful outcome
Source: F1000Res. 2016 Dec 23;5:2911. [Version 1] doi: 10.12688/f1000research.10382.1 (PMC5302217; doi:10.12688/f1000research.10382.1)
Supplement: Peer review history

# Peer review: Referee response for version 1

**DOI:** 10.5256/f1000research.11186.r18761

**Published:** 2017-02-08

**Authors:** Colin M. Howles

**Affiliations:** 1 ARIES Consulting, Geneva, Switzerland

This initiative to provide further clarity by stratifying 'Low Prognosis' ART patients into 4 main groupings is a valuable step forward in assisting clinicians and organisations considering clinical trials in such individuals.

 There is one point that I would urge the authors to consider in their future deliberations on this topic.

 If a patient in Poseidon Group 1 or 2  had a suboptimal response, following an individualised FSH dosing scheme based upon for example AMH, should this be checked again in a subsequent stimulation cycle where a higher incremental FSH starting dose is used, prior to categorising the patient in these groupings? R. Fleming has  recently referred to the existence of an 'iatrogenic poor response' (ESHRE Annual meeting 2016 Helsinki, sponsored symposium). In his dataset, a higher FSH starting dose given in a subsequent cycle (within 9 months of the poor response cycle), increased the oocyte yield. On the contrary, ART patients already identified by low AMH to be 'poor responders' did not benefit from an increase in the FSH dose.

I have read this submission. I believe that I have an appropriate level of expertise to confirm that it is of an acceptable scientific standard.

# Peer review: Referee response for version 1

**DOI:** 10.5256/f1000research.11186.r19119

**Published:** 2017-01-30

**Authors:** Ariel Weissman, Johnny S Younis

**Affiliations:** 1 IVF Unit, Department of Obstetrics and Gynecology, Edith Wolfson Medical Center, Holon and Sackler Faculty of Medicine, Tel Aviv University (TAU), Tel Aviv, Israel; 2 Reproductive Medicine Unit, Department of Obstetrics and Gynecology, Poriya Medical Center, Israel Faculty of Medicine, Bar-Ilan University, Tiberias, Galilee, 15208, Israel

Five years have passed since the publication of the Bologna criteria for the definition of poor ovarian response (POR) ^1^ and evidence is continuing to accumulate to assess their employment as well as limitations in the ART setting. This has been recently encapsulated in a contemporary critical appraisal ^2^. The POSEIDON group is making a sincere effort to introduce a more specific and higher resolution set of criteria in an attempt to guide the physician to most optimally manage this group of patients ( ^3^ and current F1000Research paper). There are, however, several reservations regarding the proposed stratification system and some clarifications that need to be made.

 Since age is perhaps the most important criterion for oocyte quality, embryo ploidy and therefore also prognosis, it is now used as a major criterion in the POSEIDON stratification, with 35 years being the age cut-off used. Thirty five years is indeed the age where both aneuploidy rates begin to rise, and pregnancy rates begin to decline in many large ART data sets. The choice of 35 years as a cut-off should be briefly explained and backed up by references.

 A large amount of research has established antral follicle count (AFC) and anti-müllerian hormone (AMH) as the most reliable and accurate ovarian reserve tests (ORT) in predicting ovarian response.  Specific cut-off values of <1.2 ng/mL and <5 antral follicles have been selected for AMH and AFC, respectively, in contrast to the less clearly defined values in the Bologna criteria, ≤5-7 follicles for AFC and ≤0.5-1.1 ng/mL for AMH. How were the cutoffs of AMH and AFC reached/decided?

 The standardization of measurement of both markers is still underway. AMH assays are in a constant process of precision improvement and automation. It is unclear for AMH whether "one size fits all", for example, should two patients, one with AMH levels of 1 ng/mL and the other with undetectable AMH levels, be counseled and managed the same way? Furthermore, currently, a 36 years old patient with regular cycles and POR, may have the same undetectable AMH levels as a 50 years old menopausal woman. Ultra-sensitive AMH levels are currently being developed, and whether they will have a role in better discrimination and prognostication of POR patients, remains to be seen. Similarly, there is yet a lack of standardization of AFC measurements. The performance and resolution of ultrasound machines is being constantly improved, and standards should be set for optimal imaging requirements. The fact that the techniques for measurement of both AMH and AFC are still under development should be mentioned.

 The POSEIDON group classifies the retrieval of 4-9 oocytes as a suboptimal response. For many, including ourselves, 8-10 oocytes is considered a goal and a successful outcome of COS, in terms of both safety and efficacy, especially in young patients.  Furthermore, Cai et al. ^4^ have recently demonstrated that similarly aged patients have similar pregnancy prospects after fresh embryo transfer when the same number and quality of embryos are replaced, irrespective of their number of oocytes retrieved. It is therefore questionable whether the range of 4-9 oocytes is again not too broad in terms of prognostication of outcome.

 The authors claim that the Bologna classification criteria have been unable to discriminate patient subsets within the POR population who could benefit from specific interventions. This is still premature to decide, as there are many studies based on the Bologna criteria that are still underway. Furthermore, a recent well performed multi-center study has shown that in normal responders the individualized treatment has similar efficacy to conventional ovarian stimulation ^5^. Whether the same is true in the POR population employing the Bologna or other criteria has yet to be explored.

   

 The POSEIDON group also suggests that stimulation should be tailored according to "the age-related embryo/blastocyst aneuploidy rate" with the intention "to retrieve the number of oocytes necessary to obtain at least one euploid embryo for transfer in each patient. The corresponding references and nomograms should be brought and constructed, respectively, in order for the stratification to become practical and for clinicians to be able to proceed with its use.

 Having an euploid blastocyst as a goal may be too simplistic, since many (in some publications >50%) euploid blastocysts never implant. Do the authors suggest to extend embryo culture to the blastocyst stage, or just use having one blastocyst as an end-point for calculation of the desired oocyte number per age group? It is questionable whether embryos in POR patients should be cultured at all to the blastocyst stage, or transferred as early as possible in the cleavage stage ^6^.  

 The POSEIDON stratification aims not only to define poor ovarian response but also to establish the prognosis for patients. It is likely that by using these criteria more homogenous populations can be established for clinical trials. However, it remains to be seen whether the management and outcome of POR patients undergoing ART can be also improved by their incorporation. It is possible that unless we will witness major breakthrough in the management of POR patients, the lay definition "women who require large doses of medication and who produce less than an optimal number of oocytes" used by patient organization Resolve ( www.resolve.org) will be as good as any stratification system.

 In summary, it is clear that the POSEIDON  group offers an improved stratification for POR patients, which has the potential to improve study designs and fine tune prognostication. It remains to be seen whether the reproductive outcome of POR patients will also be improved following the new classification system. Perhaps it is time to set an international expert meeting in order to revise the Bologna criteria and establish new consensus criteria that would enhance both diagnosis and prognosis.

We have read this submission. We believe that we have an appropriate level of expertise to confirm that it is of an acceptable scientific standard, however we have significant reservations, as outlined above.

## References

[1] FerrarettiAPLa MarcaAFauserBCTarlatzisBNargundGGianaroliLESHRE working group on Poor Ovarian Response Definition: ESHRE consensus on the definition of 'poor response' to ovarian stimulation for in vitro fertilization: the Bologna criteria. *Hum Reprod*.2011;26(7) : 10.1093/humrep/der092 1616-24 10.1093/humrep/der092 21505041

[2] YounisJSBen-AmiMBen-ShlomoI: The Bologna criteria for poor ovarian response: a contemporary critical appraisal. *J Ovarian Res*.2015;8: 10.1186/s13048-015-0204-9 76 10.1186/s13048-015-0204-9 26577149PMC4650906

[3] Poseidon Group Patient-Oriented Strategies Encompassing IndividualizeD Oocyte NumberAlviggiCAndersenCYBuehlerKConfortiADe PlacidoGEstevesSCFischerRGallianoDPolyzosNPSunkaraSKUbaldiFMHumaidanP: A new more detailed stratification of low responders to ovarian stimulation: from a poor ovarian response to a low prognosis concept. *Fertil Steril*.2016;105(6) : 10.1016/j.fertnstert.2016.02.005 1452-3 10.1016/j.fertnstert.2016.02.005 26921622

[4] CaiQWanFHuangKZhangH: Does the number of oocytes retrieved influence pregnancy after fresh embryo transfer?. *PLoS One*.2013;8(2) : 10.1371/journal.pone.0056189 e56189 10.1371/journal.pone.0056189 23457525PMC3574022

[5] Nyboe AndersenANelsonSMFauserBCGarcía-VelascoJAKleinBMArceJCESTHER-1 study group: Individualized versus conventional ovarian stimulation for in vitro fertilization: a multicenter, randomized, controlled, assessor-blinded, phase 3 noninferiority trial. *Fertil Steril*.2016; 10.1016/j.fertnstert.2016.10.033 10.1016/j.fertnstert.2016.10.033 27912901

[6] GleicherNKushnirVABaradDH: Is it time for a paradigm shift in understanding embryo selection?. *Reprod Biol Endocrinol*.2015;13: 10.1186/1477-7827-13-3 3 10.1186/1477-7827-13-3 25577140PMC4326369

# Peer review: Referee response for version 1

**DOI:** 10.5256/f1000research.11186.r19116

**Published:** 2017-01-23

**Authors:** Giuliano Bedoschi

**Affiliations:** 1 Division of Reproductive Medicine,Department of Gynecology and Obstetrics, University of São Paulo (USP) , São Paulo, Brazil

**Manuscript Review**

 The novel POSEIDON stratification of ‘Low prognosis patients in Assisted Reproductive Technology’ and its proposed marker of successful outcome

 F1000 Research

 The premise of this opinion article was to expand the discussion on the POSEIDON stratification system, including new and important information about this new concept.

 First, the authors demonstrated the researchers' dissatisfaction with the existing criteria for the definition of poor ovarian response (POR). As discussed in the article, dozens of randomized clinical trials have been published using different criteria for the diagnosis of POR. This demonstrates the importance of elaborating new stratification criteria that takes into account relevant prognosis information for POR patients. As discussed by the authors, the fact that the different strategies proposed by the clinical trials do not show improvement of in vitro fertilization cycles outcomes could be related to the dilution of the effect size when using a broad POR classification. Strategies developed for more specific groups of patients may demonstrate different reproductive outcomes. This information is of utmost relevance, especially in patients diagnosed with POR. The division of patients in subgroups would allow a more refined and individualized strategy for specific groups of patients.

 Secondly, age criterion is essential to estimate the prognosis of in vitro fertilization cycles, due to the increase in the rates of aneuploidy as the maternal age advances. The inclusion of this criterion for dividing patients’ subgroups is of great value.

 The POISEDON marker of successful outcome, based on the number of oocytes necessary to achieve euploid embryo transfer, may assist reproductive specialists for counseling purposes.

 In conclusion, the POSEIDON stratification concept presents several advantages when compared to previously described models. This facilitates the evaluation of strategies that could result in increased success of in vitro fertilization cycles for specific subgroups of patients. In addition, reproductive specialists would be able to better advise patients about their treatment prognosis.

I have read this submission. I believe that I have an appropriate level of expertise to confirm that it is of an acceptable scientific standard.
